# Affect, dis/ability and the pandemic

**DOI:** 10.1111/1467-9566.13483

**Published:** 2022-06-02

**Authors:** Dan Goodley, Rebecca Lawthom, Kirsty Liddiard, Katherine Runswick‐Cole

**Affiliations:** ^1^ iHuman, School of Education University of Sheffield Sheffield UK

**Keywords:** affect, affirmation, anxiety, dis/ability, fragility, pandemic

## Abstract

The pandemic has heightened anxieties, impacted mental health and threatened to create an overwhelming sense of existential dread. We recognise the material ways in which disabled people have been differentially impacted by Covid‐19 and make a case for understanding the affective dimensions of the pandemic. We develop a theoretical approach ‐ cutting across medical sociology and critical disability studies ‐ that understands affect as a social, cultural, relational and psychopolitical phenomenon. We introduce a public engagement project that took place in March and April of 2020 that garnered blogspots from around the world to capture the pandemic's impact on the lives of disabled people. Our data analysis reveals three key affective themes: fragility, anxiety and affirmation. To understand the emotional impacts of Covid‐19 upon the lives of disabled people we embed critical analyses of affect in the dual processes of disablism and ableism: the dis/ability complex. We conclude by considering how we might conceive of a post‐pandemic recovery that places the health and well‐being of disabled people at the centre of proceedings.

## INTRODUCTION

Over 1 billion disabled disabled people constitute 15% of the world's population (WHO/World Bank, 2011). Many people with sensory, physical and cognitive impairments are at greater risk of contracting COVID‐19, developing more severe health conditions and dying, especially if living in institutions and care homes (The United Nations, 2020b). As governments' resources strain, many disabled people struggle to access life‐saving healthcare procedures (due to health rationing and dubious notions of quality of life) and are more likely to be disadvantaged by the socio‐economic and measures to control the pandemic (United Nations, 2020a,b). These include reductions in social protection and key support mechanisms and a rise in domestic violence. UK data suggests that before the pandemic people with learning disabilities and/or autism were dying 20–30 years earlier than their non‐disabled peers and twice as likely to die from an avoidable death (LeDeR, 2019; NHS, 2017). In the midst of the pandemic, this group of disabled people were up to six times more likely to die from the virus than the rest of the UK population (Public Health England, 2020). Out of all COVID‐19 UK deaths, 59% have been disabled people, with many Black and Minority Ethnic individuals and folk with learning disabilities/autism being disproportionately impacted (Brothers, 2020).

The primary global pandemic response has been a call to shelter‐in‐place, self‐isolate and stay‐at‐home (RHJ Editorial Collective, 2020). Urban areas remain the ground zero of the pandemic, with 90% of reported cases (United Nations, 2020c). Overcrowding, rather than density, has enabled the virus to spread, with high population density actually easing the delivery of key healthcare services. Evidence indicates that tackling Covid‐19 is more difficult in urban areas with high crime rates, violence, abuse, poverty, poor infrastructure and inadequate housing (United Nations, 2020c). Shifts to remote work, and the virtual delivery of essential services have created an uncertain future for city infrastructure and buildings. The pandemic has magnified these existing inequalities; especially for disabled people. We are indebted to the work of Shakespeare et al. (2022) for their comprehensive review of the impact of Covid‐19 on the lives of disabled people. Historically, urban planning has privileged particular forms of embodied citizenship associated with a normalised non‐disabled body form (Gleeson, 1999), meaning that many disabled people experience a sense of urban displacement. Disabled people face significant challenges finding suitable housing (EHRC, 2018). Those that were able to stay‐at‐home did so in poor‐quality homes; exposed to cold, damp and other hazardous conditions with consequences for physical and mental health. Housing conditions are typically poorest for Britain's 5.5 million private rented sector households. In 2019, one‐quarter of disabled people lived in rented social housing, compared with just 8.2% of the general population (ONS, 2019). Personal Protective Equipment (PPE) was provided for hospitals, but not for care homes, and then in care homes but not for home carers and personal assistants (Shakespeare et al., 2022). Inequalities are evident in people's capacity to comply with social distancing measures, with this being most difficult for those on low incomes, in insecure employment and living in overcrowded homes (Shakespeare et al., 2022). Over 60% of disabled people struggled to access food, medicine and necessities; including inaccessible websites; lack of on‐line delivery slots and confusing guidance and information. Many supermarkets and shops failed to make reasonable adjustments such as support for people who cannot queues or reach items (Inclusion London, 2020). Public transport systems around the world have seen ridership and revenue plummet and have been forced to cut services (United Nations, 2020c), leaving many disabled people immobile.

Long‐standing fundamental weaknesses in public services have been exposed and health inequalities magnified by the pandemic (Parliament UK, 2020). Article 11 of the Convention on the Rights of Persons with Disabilities obliges governments to take all necessary measures to ensure the protection and safety of disabled people in situations of risk, including situations of humanitarian emergencies and the occurrence of natural disasters. Yet, Do Not Resuscitate (DNR) orders have been imposed with little or no consultation and disabled people have been denied routinely planned medical treatment (Inclusion London, 2020). Organisations of disabled people, including self‐advocacy groups of people with learning disabilities and/or autism, have shifted online (Shakespeare et al., 2022). Many charitable organisations reported that their fundraising income had reduced substantially ‐ caused by closure of charity shops, lack of street collections and fundraising events (Parliament UK, 2020). At the same time, there is also evidence of increased reliance on their family and other informal carers, driven by the closure, or suspension of day centres, day services and large sections of the social care system, and large numbers of social care contracts were cancelled, put on hold, or severely limited (Shakespeare et al., 2022). Disabled people have expressed concerns about having too many people coming into their own homes and wanted to reduce contact. As a result, where it was possible, they preferred using family members who were already part of ‘their social bubble’. There is concern about the impact this may have on the security and stability of their care in the future (Shakespeare et al., 2022, p. 11). Disabled people's access to democratic forms of engagement are hugely impacted by their experiences of work. The unemployment rate for disabled people is 6.5% (this is compared to 3.5% for non‐disabled people) (House of Commons Library, 2020). Leonard Cheshire (2020) found that 7 in 10 disabled people, employed in March 2020, have been impacted by: loss of income, furlough or unemployment; 42% of employers were reluctant to hire disabled people due to concerns about offering support during the pandemic; 1 in 5 employers (20%) say they would be less likely to appoint a disabled applicant; 18–24 year old disabled people were worried about their future earning potential and the impact on their mental health. Evidence suggests that there have been some improvements in working conditions for disabled people due to the shift to online ‐ no longer needing to navigate inaccessible buildings and public transport. Digital exclusion disproportionately impacts disabled people 56% of adult Internet non‐users are disabled, while only 22% of the UK population are disabled (ONS, 2019). Many disabled people, like others, are experiencing the stress of working from home, caring, and home‐schooling (Shakespeare et al., 2022). Employers are failing to introduce reasonable adjustments to enable Deaf and disabled people to work from home (Inclusion London, 2020).

For disabled children and young people, the closure of schools has meant a loss of access to inclusive and specialist forms of education; with only a few benefitting from online or distance learning (United Nations, 2020a, b; Shakespeare et al.,  2022). Disabled young people with underlying health conditions (those at greater risk) have struggled to access life sustaining treatment and therapies, including mental health services, during school closures (Children and Young People's Commissioner Scotland, 2020). OfSTED (2020) has recognised a crisis in mental health, wellbeing and learning. The Coronavirus Act 2020 temporarily amended the absolute duty to make the provision in an Education, Health and Care Plan (EHCP) (Children and Families Act, 2014) to a ‘reasonable endeavours’ duty. This meant that local authorities were required to do whatever they reasonably could to meet EHCP, but if they could not do so they would not necessarily have been breaching the law (IPSEA, 2021). The move from classrooms to online learning/working environments has excluded some disabled people as they are pulled away from established forms of assistance and pedagogical support. Certain technologies are inaccessible and this adds to a state of affairs where many are already digitally excluded (especially those in lower socioeconomic groups). The special school sector has been ignored by the government which has shown a lack of understanding of how special schools work, the types of pupils they support and how much they rely on other services, including health and social care, transport and local charities (Skipp & Hopwood, 2020). Special schools report that children and young people are unable to access community based learning, volunteering and work experience which is part of their core curriculum (OfSTED, 2020).

Our brief literature reveals significant material impacts on the lives of disabled people but is also limited. Firstly, our Anglocentric review clashes with realities of a global pandemic. One of the knock‐on effects of social restrictions has been a reaffirmation of banal nationalism and banal localism (see Aiello & Kennedy, 2021): a sense that the main issues of concern are those that are (only) close to home. It is perhaps inevitable that lockdown makes people more parochial. Nevertheless, we want to extend a more pan‐national understanding of the interrelations between dis/ability, affect and Covid‐19. While our analysis is not comparative ‐ nor representative of all national contexts ‐ we do seek at least to offer some snapshots of contemporary life in different national spaces. Secondly, questions remain about the emotional impacts of Covid‐19; a turbulent two years that have heightened feelings of anxiety, fear and uncertainty. And it is this affective register that forms the focus of our analysis. We are interested in theorising the ways in which affects are mobilised by economic, social and cultural forces in the lives of disabled people (Goodley et al., 2018). We ask: what are some of the emotional impacts of the pandemic on disabled people across a host of national contexts? How might we theorise these affective realities? To address these questions we engage with responsive social theories.

## THEORISING AFFECT

A large body of research and knowledge has been generated around the psycho‐emotional impacts of Covid‐19. Tyrer (2020) argues, from a mainstream psychiatric perspective, that the pandemic has undoubtedly led to a host of ‘pathological health anxieties’ (Ibid). Similarly, from a health psychology perspective, research has already found a positive association between both generalised anxiety and COVID‐19 specific anxiety and the reporting of somatic symptoms such as gastrointestinal and fatigue symptoms (Shevlin et al., 2020). Similarly, Rudenstine et al. (2020) report on increased ‘depressive and anxiety symptoms’ amongst young people living in the COVID‐19 epicentre of the U.S. pandemic. It is important we pause here for a moment to sit with the contemporary cultural moment; a time where Big Pharma, health systems and medical expertise are lauded for the creation of vaccines and the provision of life‐saving treatments. We have no sense yet of the extent to which the practices of medicalisation (Busfield, 2017), psychologisation (Vos, 2012) and psychiatrization (Mills, 2014) are being replenished, recuperated or even possibly mutating as a consequence of research in response to Covid‐19. What we can state is that when pandemic anxieties and emotions are treated as individualised problems ‐ explained in ways that emphasise biochemical, psychical or organic factors that are firmly rooted in the psychologies and bodies of their ‘sufferers’ ‐ then there is an urgent to intervene with social and cultural critique.

Our paper feeds into what Wetherell (2015) describes as the reworking of the heartland of sociological topics through the lens of emotion and affect (see Goodley et al., 2018 for a detailed overview). More researchers are engaging with what Wetherell terms psychic landscapes, passions and feelings of human life that have, at times, been ignored or rejected by the more structural or discursive tendencies of sociology. The affective turn is a popular and broad term that risks simplifying and generalising a highly disparate field of research that spans anthropology, cultural studies, education, sociology and critical psychology. In our brief reading of the affective turn we consider three broad areas of theorisation that set the foundations for our analysis of affect, dis/ability and the pandemic.

We are interested in engaging with the social and cultural foundations of affect. We are influenced in particular by the work of Ahmed (2004, 2007/2008, 2010) who attends to the ways in which different *affect economies* circulate around and impinge upon emotional life. Anxiety and fear are experienced as embodied or psychical experiences but have histories to them that are reproduced through various socio‐cultural practices. Neilson (2015), for example, demonstrates how various iterations of neoliberal capitalism have produced specific kinds of precarity that are experienced or felt by individuals in very different ways. Job insecurity, the imperative for self‐sufficiency and the retrenchment of welfare services increase individual's sense of their own precarity and these material conditions provide a fertile breeding ground for the production of anxiety. We concur with Ahmed that emotions manifest themselves in profound ways dependent upon place and space marked by inequality and opportunity. Emotions and sensations (such as those associated with anger, discomfort or elation) are often experienced as nebulous visceral, instinctive or primitive feelings (Wetherell, 2015). However, while we might *feel* emotions, they are situated in human and non‐human relations. New materialist sociologies have sought to ‘turn away from individualistic and anthropocentric emphases upon the experience of feelings and emotions, attending instead to an exploration of flows of ‘affect’ between bodies, things, social institutions and ion (Fox, 2015, p. 301). Rather than understanding affect in terms of psychoanalytic instincts or biopsychological urges, new materialists pitch an understanding of affect as a phenomenon created in the dynamic interrelationships between humans and their environments (Fox & Alldred, 2015).

While social, cultural and relational approaches attend to the ways in which a person's emotional life can never be separated from their material or political circumstances, psychopolitical interpretations of affect go one stage further: by considering the ways in which power is wielded over and through the psyche. Psychopolitical perspectives interrogate how ‘politics impacts upon the psychological’ and how a person's ‘psychology may repeat, internalise and further entrench such political effects at the level of personal identity’ (Hook, 2004, p. 90). Fanon's work (1976) is often associated with the foundations of psychopolitics. He sought to comprehend and resist the ways in which white privilege and colonial practices threatened to pathologise the subjectivities of colonised people. Any affective response ‐ whether it be denial, fear, guilt, depression or anxiety ‐ has to be contextualised in a social context in which black people are often constituted as being deficient, lacking or absent (see He, 2020). Psychopolitics understands affect as entangled with material and historical conditions associated with class, gender, sexuality, race and disability (Cresswell & Spandler, 2009). A psychopolitical analysis seeks to embed any interrogation of anxiety in the wider socio‐political context in which the bearer of those emotions is located (Mills, 2018).

## THEORISING DIS/ABILITY

To expand upon these socio‐cultural, relational and psychopolitical understandings of affect we turn to the experiences of disabled people. We understand disabled people as those individuals living with sensory, physical or cognitive impairments. Comprehending their affective pandemic lives necessitates an interrogation of the ways in which society and culture understands and values disabled people. This leads us to critical disability studies (Meekosha & Shuttleworth, 2009; Mallett & Runswick‐Cole, 2014). While disabled people are far from a homogenous group, disability scholars and activists have identified some common ideological encounters. One of these is disablism which Carol Thomas's (2007a: 73) defines as ‘a form of social oppression involving the social imposition of restrictions of activity on people with impairments and the socially engendered undermining of their psycho‐emotional well being’. The other ideological encounter commonly experienced by disabled is ableism, which can be understood as the material, cultural and political privilege of ability, sanity, rationality, physicality and cognition (Goodley, 2014). Ableism and disablism feed off each other; they are coterminous. One of us (Goodley, 2018, p. 7) has suggested that it might be helpful to think about the pull and push of these two processes ‐ ableism and disablism ‐ as constituting the dis/ability complex. This complex creates a bifurcated reality ‐ just as disability is diagnosed so ability is further expanded ‐ as society holds more sway in the promises of self‐sufficient, autonomous, and able citizens so those that fail to meet up to the ableist zeitgeist are rendered disabled (Goodley, 2018). We are interested in understanding the pandemic's socio‐cultural, relational and psychopolitical constitution of the affective lives of disabled people and to consider the ways in which the dis/ability complex plays out.

In recognition of this special issue, we want to bring critical disability studies perspectives to the fore in an empirical and theoretical context where medical sociology often prevails. Clearly, the health and well‐being of disabled people is of interest to medical sociology. For critical disability studies, however, disabled people's health and well‐being are deeply personalised and politicised. As Carol Thomas observed (2007a, b) and Gareth Thomas reiterated later (2021), medical sociology and critical disability are in danger of passing one another like ships in the night. Medical sociology has tended to talk *around* rather than *about* disability (preferring analyses in terms of social deviance) and critical disability studies situating understandings in terms of social oppression (very much influenced by social and materialist models of disability). While more contemporary scholarship has brought together the two perspectives (e.g, Mauldin & Lewis, 2021; Thomas, 2021) we note that there are often tensions between these two fields of scholarship. In particular, critical disability studies occupy a liminal space between academia/activist and theory/praxis. This position is due in part to critical disability studies' origins within the politicisation of disabled people and the generation of their knowledge. Disability is the *driving subject* of inquiry. In medical sociology, disability has been largely conceptualised as an *object* of inquiry: as researchers and theorists draw in disability from its historically peripheral place on the edge of their intellectual and empirical communities. While our epistemological, ontological and methodological home is very much in critical disability studies we should acknowledge our medical sociological predilections. The very title of our paper objectifies disability. Our writing attends to disability as a phenomenon worthy of analysis. One might view the discursive moves in this paper ‐ from disability, to affect, to the pandemic and back again ‐ as a journey around disability. When we shift gears, to engage with the demands of critical disability studies, we find disabled people's experiences, aspirations and expertise driving our analysis. In the turn to affect we find an intellectual space in which a *frictional relationship* between medical sociology and critical disability studies might be forged. This concept, borrowed from Puar (2012), encourages an analysis of the ways in which health and well‐being (staple topics of analysis for medical sociologists) are deeply impacted by processes of ableism and disablism (conceptualised by critical disability studies) in the context of Covid‐19.

## METHODOLOGY AND ANALYTICAL APPROACH

We offer a broad thematic analysis of emerging themes drawn from a public engagement project coordinated and hosted by iHuman (the Institute for the Study of the Human) at the University of Sheffield. In the weeks following the first UK lockdown in March and April of 2020, members of iHuman sought to collate information from Britain and other countries that captured some of the ways in which disabled people were being impacted by the pandemic. This produced the crowd‐sourced resource Disability and Covid19: The global impacts. Blogs have been established as a source of data and research methods in their own right across the social and human sciences (e.g. Keim‐Malpass et al., 2014). The blogs we collected provided partial qualitative snapshots, auto‐ethnographies, opinion pieces, reflections on data, narratives and critical commentaries. Bloggers offered descriptions and analyses of their national contexts; stark reminders of the conditions of oppression that many disabled people endured before and during the pandemic. It is important to acknowledge enduring socio‐historical, economic, political and economic realities that threaten to dehumanise disabled people before, during and after the pandemic. It was only through a close reading of the pieces that we found affect to be omnipresent.

As Hookway (2012) notes, moving into the blogosphere offers research possibilities and raises numerous ethical issues. Crucially, this sphere opens access to researchers and activists who would otherwise be geographically or socially removed from one another (Ibid). This was definitely the case with our call for contributions which was emailed across a host of social media and distribution lists. In all, 22 contributions were received from 15 countries and uploaded as a series of blogs, written by researchers, activists, disabled and non‐disabled people. All the blogs used in this paper were submitted to one or more of the authors of this paper who were responsible for the website that houses the blogs. In some cases contributions were returned to authors to address minor typographical and grammatical errors or to seek more information (for example full references or work cited). None of the bloggers were asked to change the tenour or tone of their pieces. We did not consider fictionalising nor anonymising the work. Our view was that these blogs constituted important voices from the field and maintaining ownership was crucial. We have sought in this paper, wherever, possible, to cut and present extended direct quotations from the blogs. This permits the nuance and power of contributions to sit within the wider argument that we, as the authors of this paper, take ownership. We have also, at times, summarised some of the bloggers' key arguments, observations and assertions.

Our analysis seeks to speak across the bloggers' contributions and we take full responsibility as authors for any analytical failings. Our analysis deploys a broad thematic analysis approach drawing upon established ideas in the social science literature (Braun & Clarke, 2006; Bannister et al., 2011) supplemented by an openness to the interplay of deductive and inductive approaches. We acknowledge that our interests in socio‐cultural, relational and psychopolitical theories of affect ‐ alongside a critical disability studies engagement with processes of ableism and disablism ‐ have driven the more deductive aspects of our analysis. We would accept that, in part, we found themes because we looked for them. We have also sought to engage with an inductive approach too; ensuring that our data set has helped shape the resultant themes. The inductive aspects of our analysis were driven by attending to the specifics and the singularities of each blog. While this happens with any inductive approach ‐ data driving analysis ‐ blogs have even more persuasive quality to them. Each blog constitutes a scholarly online research publication in its own right while many of them combine evidence with conjecture. Contributions are referenced as publicly available works, with many centralising disability and the lives of disabled people in their analysis of inequity and social justice. The inductive aspects of our analysis were also informed by the strong positionalities of many of the contributors: in which they called for social change and action. During the writing of analysis we cited the blog contributions (as we would any already published work) and we felt engaged with a community of scholars and activists.

## ANALYSIS

We lay out below three themes: fragility, fear and affirmation. In writing analysis we are thinking of the ways in which our discussion cuts across medical sociology and critical disability studies in order to theorise affect as a social, cultural, relational and psychopolitical phenomenon. And in seeking to understand the emotional impacts of Covid‐19 upon the lives of disabled people we embed our analysis of affect in the dual processes of disablism and ableism: the dis/ability complex.

### Fragility

The pandemic has globally engendered feelings of fragility. For disabled people, however, this emotional reality has been shaped by a dominant cultural imaginary in which their very human worth has been called into question. The vast majority of our blogs engaged with the ontological insecurities of disabled people. Liddiard (2020)'s blog captures the tone of some of the early responses to the pandemic by the British government. These included appeals to herd immunity; the notion of allowing publics to be exposed to a virus, in the hope that spreading it among those who are at low risk means that a large part of the population becomes immune. Whilst this approach was quickly quashed in the UK, its reliance upon dangerous Social Darwinist ideas suggested something more problematic at the heart of government. Altermark (2020) argues, from a Swedish context, that the function of ‘risk groups’ is to reassure ‘normal’ people that someone else will die. The trope of at‐risk groups works, he writes, ‘to restore the myth of self‐sufficiency and self‐mastery by placing risk somewhere else, with the elderly, the disabled, and those with chronic illnesses’. This discourse helped support the Swedish government's strategic emphasis on individual responsibilisation over state intervention ‐ the concept of folkvett ‐ the common sense of the people as a collective (Orlowski & Goldsmith, 2020).

Here we can identify cultural work being down either side of the dis/ability complex (Goodley, 2018): where disabled people are assigned an at‐risk categorisation (which may or may not be recognised in discussions of herd immunity) while non‐disabled people are assumed to be healthy (which actually fails to recognise the precarity of an able‐bodied and minded status). A similar framing of dis/ability is reported in Schippers' (2020) blog from the Netherlands with worries being expressed by disabled people about a triage system that prioritises ‘otherwise healthy lives over vulnerable and disabled lives’. The constitution of disabled people as fragile subjects took on a particularly dangerous turn in the early stages of the first lockdown through the use of the Clinical Frailty Scale in the UK. Liddiards (2020) blog contribution understands this as a ‘troubling measurement of human worth and value’ with regulatory organisations such as the National Institute for Clinical Excellence (NICE) utilising this scale as a key mechanism for rationalising admission to critical care in the pandemic. ‘In short’, Liddiard writes, ‘a score of 5 or below makes you more likely to access critical care, while a score of 6 or above makes this less likely. Medical professionals must ‘take into account the impact of underlying pathologies, comorbidities and severity of acute illness on the likelihood of critical care treatment achieving the desired outcome’ (2.2 NICE Guidelines, March 2020).

When systems are under strain then guidance around healthcare rationalisation, like that associated with the Clinical Frailty Scale in the UK, are used to justify governments' prioritising the treatment of non‐disabled bodies with Covid‐19 over disabled bodies (Núnez‐Parra et al., 2020). This observation is supported by Kritfunk's blog (2020) who report on intensive care guidelines in Sweden focussing on treating those with ‘the greatest chance of survival’ thus placing ‘ill and disabled people, regardless of age and other health factors, as collateral damage’. Ignagni et al. (2020) push this point further, in their blogpost, asserting that disability equates with expendability evidenced in Ontario, Canada's proposed medical triage protocol regarding COVID‐19 medical attention. And similarly, in Greece, health care rationing and the constructed criterion of ‘quality of life’ risk the application of guidelines that reflect principles of eugenics: separate those who are worthy to live from those whose lives are not valued (see the blog by Tsakiri et al., 2020).

Moreover, as Amber (2020) puts it in her blog: receiving a letter from her local GP outlining her health status shifted her position overnight from being a proud disabled person to being a recognised vulnerable member of society. The re/constitution of disabled people as vulnerable threatens to feed the dominant cultural logics of the dis/ability complex: devaluing disability (disablism) while simultaneously and unproblematically upholding ability (ableism). As Ktenidis (2020) blogs, these logics create forms of ontological violence, precisely because ‘the deaths of those belonging to the ‘vulnerable’ groups were deemed natural and, hence, mattered less, in opposition to the deaths of people who were not vulnerable, and, therefore, their death was ‘unnatural’ and mattered more’.

From a psychopolitical stance, therefore, the message is clear: abled lives are worth saving while disabled lives are expendable. The affective registers of disabled and non‐disabled people are flooded with signs, meanings and discourses that threaten to create a feel of disability as disposable; as worthless subjects (sidelined in considerations of herd immunity) and worthless patients (disqualified from receiving primary care). The intimate impact of this situation is captured in Haraldsdóttir's (2020) writing from Iceland; ‘I am a disabled woman in a body that is labelled as weak and unworthy – disposable’. Unlike most fragile items ‐ which we are also counselled to ‘Handle with Care’ ‐ disabled bodies risk being made disposable; cast as waste products of a healthcare system that prioritises the recyclable. From a psychopolitical perspective, then, *feeling fragile* is exacerbated by a dis/ability complex that values (assumed) ability over (prescribed) disability.

### Anxiety

Gahatraj (2020) blog written from Nepal makes the case that COVID‐19 is not just a health pandemic but it is also socio‐economic and cultural pandemic as people's lives have been put into crisis. ‘There are’, they argue, ‘two aspects of direct effect on the lives of persons with disabilities – one is being more anxious of its life‐threatening impacts and another is hardship created by extended countrywide lockdown’. It is important that we recognise and seek to understand the heightened fears and anxieties of disabled people not as symptoms of some underlying psychopathology but as affective reactions to the disabling consequences of the pandemic. Amber's (2020) blog details the first lockdown in England, which created huge pressures on the availability of online shopping and safe public transport, with these material factors impinging massively on well‐being. Kritfunk (2020) reports on the psychological impact on disabled people in Sweden caused by poor experiences of online communication (due to inaccessible design of devices, platforms and content) and an increased sense of isolation (caused by stay‐at‐home and social distancing). Pilson (2020) attacks what she terms ‘herd accessibility’ endemic in an ‘assumed equality of digital access’ and a ‘blasé approach to digital literacy’ that has moved us to a new normal of face‐to‐face and synchronous meetings without any thought being given to the potential for exclusion. We know, too, that many disabled people have lost key services provided by non/government organisations (Spence, 2020). Disabled people are, according to (Ignagni et al. (2020), ‘navigating the uncertainties of virtualising care by transferring carefully crafted support arrangements to the telephone and video conference’. Anxiety and existential dread are bedfellows; ‘Our situation, now more than ever, is tangled up in how and if someone will catch us if we fall’ (Haraldsdóttir, 2020). Disabled people risked being plunged to ever more anxious moments as online and offline worlds became more inaccessible (disablism) just as they emphasised self‐sufficiency (ableism). The sheer anxiety‐provoking realities of the dis/abiltiy complex are captured further by Van Hove (2020), blogging from Belgium, who writes about the ways in which disabled people and their families were thrown into the catastrophe of Covid‐19 after decades of systemic neglect:

Many families were faced with the ravishing choice: leaving their relatives in an institution (knowing that these are possible ‘dead houses’ during a health crisis) or taking care of their children / relatives themselves (knowing that the home care service providers are not sufficiently trained to share the support) (Van Hove, 2020).

Karagianni (2020) writes from a Greek context noting how public discourse around the pandemic failed to recognise disabled people; focussing only on ‘the aged and the ill’. Lockdowns and social distancing have been key strategies in the fight against Covid‐19. Pilson's (2020) blog recognises the anxieties associated with visually impaired and blind people in adhering to ideas of social distancing. She writes, ‘social distancing is a visually dialogic process that automatically absents visually‐impaired people from possessing equality of power in public places’. Similarly, considering the Canadian context, Ignagni et al. (2020) write:

…for many of us care still depends on physical touch. Where is the guidance on supporting disabled people to use PPE and maintain social distancing? In the public health direction around the use of masks and gloves, there has been no guidance on how to safely mask a disabled person.

These challenges are picked up on by Gahatraj (2020) writing from Nepal who notes that social distancing has broken established care and support relationships. The loss of support undoubtedly impacted on feelings of isolation and anxiety. Meanwhile in Latin America, many disabled people who have historically experienced abject poverty and the lack of social protection have been plunged into further uncertainty (Grech, 2020). These inevitably impact on the intimate daily lives of disabled people:

The primacy of touch and visceral interdependence in our everyday survival that allows us to keep our balance, find our way, or get out of bed, places us in complicated and contradictory relation with the notion of social distance. (Ignagni et al., 2020).

Interdependence takes on many forms. Grech (2020) describes how social lockdowns have prevented life‐saving acts of charity or informal family support in Guatemala. Moreover, for those disabled people exempted from mask‐wearing or strict lockdown measures (on the grounds of impairment), this has created a number of troubling emotional encounters:

‘Irresponsible! You put all of us in danger! Shame on you! Die!’ These are some of the comments that autistic people and their parents have been hearing from balconies and windows of their own neighbourhoods, even after the [Spanish] government legally recognised their right to be in the street during the emergency state (Monforte & Úbeda‐Colomer, 2020).

These impairment‐related exemptions from staying‐at‐home (as in the case of Spain) or masking (in the case of the UK) risk being misunderstood in their social contexts because of expectations around particular kinds of normative behaviours that are markers of responsible citizens. It is not simply the case that citizens have to take responsibility for their own care and protection; they are expected to do so in socially prescribed normative ways. As Schippers (2020) writes, ‘if individuals, with or without disabilities, become deadly ill by the virus, it is they who are to blame, because they did not practise proper social distancing’. Hence, while many of us experience anxieties when encountering environments outside the safety of our homes, these worries are multiplied for disabled people when their behaviours risk being interpreted as irresponsible. We might understand these circumstances as the psychopolitics of neoliberal‐ableism (Goodley, 2014): where anxieties are magnified by feelings of failure to fit socially prescribed normative expectations associated with self‐containment and responsibilisation as the key responses to the pandemic.

### Affirmation

One positive development of the pandemic was the surge in civic activity. By late 2020, over 4000 mutual aid groups had emerged in the UK during the pandemic and there is evidence to suggest that they have reached out to ‘hard to reach groups’, including disabled people (Parliament UK, 2020). Many of the blogs reflected on these emerging communities. Schippers' (2020) blog suggests that we have seen the emergence of new kinds of solidaristic relationships in the Netherlands. One should not underestimate the affective impact of these affirmative relations, especially in times where disability is too easily associated with deficiency and tragedy. Disability communities have, of course, long histories of generating bonds and relational systems of support that emphasise what Liddiard (2020) blogs as emphasising ‘human worth, value, and desire for the future’. Disability communities have collective memories about the damage done by disablism and ableism and, in response, create new positive affect economies that circulate through and around disabled people (see Ahmed, 2004). One productive consequence of the shift towards online participation, triggered by the pandemic, was that many disabled people were able to participate in group activities that had previously been denied to them because of physical barriers and inaccessible infrastructures (Ryan, 2020). While disabled people undoubtedly experience digital exclusion (Macdonald & Clayton, 2012), Sunderland People First's (2020) blog evidences the increased opportunities for daily contact offered by the deployment of their online community outreach. Similarly, Sheffield Voices (2020) at Disability Sheffield in England, has deployed zoom coffee mornings to connect with people with learning disabilities across the city.

Living through the difficult times of the pandemic is associated with having a voice and feeling more included. Feeling better is not simply about feeling healthy but also about feeling included, supported and recognised. This responsive and empowering work by self‐advocacy groups captures what Roets (2020) blogs as ‘people who receive and give care’ making ‘the best of their circumstances, fuelled by commitment, social imagination, hope and solidarity’. Speak up Self‐advocacy Rotherham (2020) told us that they have also set up regular zoom meetings for people with learning disabilities that they support. This is crucial because, as they write, ‘Our communities of interest are people with learning disabilities, autism or both, many of whom have enduring mental health conditions. The current ‘lockdown’ is causing people's anxieties and levels of crisis to increase significantly’ (Speakup, 2020). Due to COVID‐19, peer‐led interventions are providing crisis support and one to one support with mental health and wellbeing through daily zoom calls, online one‐to‐one sessions who are isolated of feeling depressed and group support sessions in the form of quizzes, mindfulness, massage, yoga, talking therapy, cooking and life skills which people can join – run by self‐advocates. Other online connectivities are detailed in Pilson's (2020) blog: VIEW has collated an exhaustive list of resources for home‐schooling and leisure; VICTAR has extended grants for assistive technology; Look UK has created online fora and webinars to combat social isolation. This in turn has been echoed by individuals on social media, for example, by the creation of virtual socialising spaces like the Staying Inn on Twitter (@TheStayingInn).

Speakup (2020) urge us to historicise anxiety: to recognise that people with learning disabilities were already living precarious lives and that their mental health cannot be bracketed from wider social and cultural practices. The affirmative potential of disabled people's activism is picked up on by Karagianni (2020) who describes the Greek ‘disabled activist group ZERO TOLERANCE since the beginning of the covid‐19 crisis in early March has brought to fore, with contributions to newspapers and networks, the situation of the disabled (who are poor, low paid, unemployed, elderly, school age children, asylum residents, refugees, women and people living alone in the community) promoting a broad understanding of a group combating with the ‘multidimensional oppressive matrix’ (Humphrey, 2000).

Bloggers were keen to share with us ways of connecting that promoted empathy, dignity, compassion, kindness and solidarity.^1^ As one of our bloggers writes: ‘the Covid‐19 era is developing into a historical moment that lends itself to reflection, acts of solidarity and opportunity for change’ (Karagianni, 2020). While it might be useful to draw on new materialism to understand these dynamic interrelationships between humans and online environments in terms of assemblages (Fox & Alldred, 2015) we are keen to also acknowledge the affective potential of these connections. We might read these as emerging psychopolitical spaces of affective support, affirmation and recognition. And many of these online spaces have a very humanising quality to them; contrasting with the deeply dehumanising experiences of pandemic life.

## CONCLUSION

In this paper we have engaged with disabled people's experiences, aspirations and expertise in relation to some of the affective dimensions of the pandemic. We have sought to demonstrate the analytical possibilities of creating an intellectual space in which a *frictional relationship* between medical sociology and critical disability studies might be forged. Throughout the reading of affect we have sought to keep in mind the dis/ability complex (the simultaneous pull and push of ableism and disablism) and have argued for a theorisation of affect that is social, relational and psychopolitical. While disabled people's health inequalities have been magnified by the pandemic, we concur with Lightfoot (2000a, b) that ‘the current post‐covid period of recovery offers a once‐in‐a‐generation opportunity to build a society that works for everyone’. This opportunity has been recognised across supra/national policy. The United Nations themed the 2020 Day of The International Day of Persons with Disabilities (IDPD) as Building Back Better: towards a disability‐inclusive, accessible and sustainable post COVID‐19 World (United Nations, 2020a, b). The United Nations (2020b) ‘A Disability‐Inclusive Response to COVID‐19’ Policy‐brief published in May 2020 outlines a number of key recommendations including mainstreaming disability in all response and recovery programs, consulting with disabled people and their representative organisations and ensuring that governments, donors, United Nations agencies and other actors establish mechanisms to monitor investments to ensure they are reaching disabled people. This emphasis on a ‘disability‐smart’ future is shared by The World Bank (2020) which asserts that an inclusive post‐pandemic recovery for disabled people should focus on: ready access to the vaccine; inclusive infrastructure and digital inclusion; access to education, health and sanitation and real opportunities for work and building long‐term work skills. One could argue, that at least rhetorically, disabled people are being foregrounded in a number of policies associated with the post‐pandemic recovery. Central to any programme of recovery is the need to consult with community stakeholder groups. Despite the increased risks disabled people face as a result of COVID‐19, their experiences are often missing from reports published during the pandemic and they have been excluded from wider decision‐making processes (Shakespeare et al., 2022). One of our bloggers Barod (a workers cooperative for people with learning disabilities), makes a strong case for the central involvement of disabled people in research:

[A] lot of academics are currently rushing to do research about COVID‐19. We have a message for you: Please don’t try to do it on your own. You need to make sure people whose lives will be affected are part of shaping what you are thinking, planning and doing’ (Barod, 2020, np).

We align ourselves with another of our Canadian contributors Underwood and Parekh (2020) in their hope that our current ‘care relationships that make us human will be remembered and valued, in addition to containing the virus and re‐starting the economy’. Key to recovery plans is the need to ensure that we understand the affective dimensions of the pandemic in cultural and political ways. Our analysis has revealed the ways in which people (living either side of the dis/ability complex) are deeply impacted by wider discourses of human worth and disposability. The bloggers represented in this paper qualitatively capture some of the differential ways in which nation states and governments have responded to the plight of disabled people. The dangers of supranational discourses that merge considerations of dis/ability and the pandemic is that they might fail to connect with the local realities and priorities of disabled people. We need to be in tune with these local and national circumstances whilst being wary of falling into banal nationalism. Conceptions of health and well‐being in the lives of disabled people must always be informed by a critical sensibility; thus recognising that affective touchpoints of fragility, anxiety and affirmation are always under‐girded by wider questions of disability and humanity. Moreover, these questions are always historically, culturally and socially located; dis/ability is felt very differently in and across diverse contexts. Critical disability studies have much to offer in this regard. Pescosolido and Kronenfeld (1995, p. 9) write that ‘sociology holds its greatest appeal in times of disarray’. The same could be said now of medical sociology as researchers seek to make sense of the emotional and material impacts of the pandemic. What is required, however, is the frictional demands of critical disability studies: medical sociology must attend to the differential impacts of disablism and ableism on the lives of disabled people.

## AUTHOR CONTRIBUTION

Drafting and revising of manuscript and study design Professor Dan Goodley, Professor Rebecca Lawthom, Dr Kirsty Liddiard and Professor Katherine Runswick‐Cole. Obtained funding Professor Dan Goodley, Professor Rebecca Lawthom and Professor Katherine Runswick‐Cole.

## Data Availability

The data used in this paper is available online and open at https://www.sheffield.ac.uk/ihuman/covid‐19‐blog.

## ENDNOTE

^1^
These ideas of empathy, compassion and humanisation form the focus of a new Economic and Social Research Council funded project Humanising the Healthcare Experiences of People with Learning Disabilities and/or Autism (ES/W003406/1). The form, content and value of humanising forms of care and contact have taken on a particular salience as a consequence of Covid‐19.
